# What to do with K-wires in displaced upper extremity fractures in children – where to bury or not

**DOI:** 10.1007/s00068-026-03353-8

**Published:** 2026-09-29

**Authors:** Andreas Frodl, Marietta Borbely, Nikos Karvouniaris, Nils Mühlenfeld, Markus Siegel, Kerstin Kuminack, Carlotta Pietsch, Ferdinand C. Wagner, Benjamin Erdle, Hagen Schmal

**Affiliations:** 1 Department of Orthopedics and Traumatology, Freiburg University Hospital, Freiburg, Germany; 2 Department of Orthopedics and Traumatology, St. Josefs Hospital Freiburg, Freiburg, Germany; 3 Department of Orthopedic Surgery, University Hospital Odense, Sdr. Boulevard 29, Odense C 5000, Denmark

**Keywords:** Kirschner wire, K-wire, Pediatric fractures, Upper extremity, Infection, Buried, Exposed, Systematic review, Meta-analysis

## Abstract

Kirschner wires (K-wires) are the standard fixation method for displaced pediatric upper extremity fractures, yet no consensus exists on whether wire ends should be buried subcutaneously or left exposed epicutaneously. While epicutaneous K-wires offer cost-effectiveness and avoid second anesthesia, concerns remain regarding infection risk and psychological distress. To systematically review and compare complication rates between buried and exposed K-wire fixation in pediatric upper extremity fractures, including infection, refracture, skin erosion, and secondary implant displacement. We conducted a systematic review following PRISMA guidelines, searching Cochrane, PubMed, EmBase, and Ovid databases. Inclusion criteria were: modified Coleman Methodology Score (mCMS) > 60, upper extremity fractures treated with K-wire fixation, and age < 16 years. Studies including adult patients and biomechanical studies were excluded. Meta-analysis compared outcomes across 12 studies encompassing 1,977 patients. Buried K-wires demonstrated significantly lower overall infection rates (OR = 1.74; 95% CI: 1.19–2.56; *p* = 0.004). However, no significant difference was observed in deep surgical siteinfections (*OR = 1.56; 95% CI: 0.60–4.0; p = 0.36*). Exposed wires showed significantly lower skin irritation rates (OR = 0.09; 95% CI: 0.04–0.21; *p* < 0.00001). No significant differences were found in secondary implant displacement (OR = 1.26; 95% CI: 0.10–15.75; *p* = 0.86) or refracture rates (OR = 1.90; 95% CI: 0.91–3.96; *p* = 0.09). Geographic subgroup analysis revealed significant regional differences: infection rate differences were significant in Asia/Africa (OR = 2.90; 95% CI: 1.61–5.22; *p* = 0.0004) but not in Europe/USA (OR = 1.16; 95% CI: 0.69–1.95; *p* = 0.57). Epicutaneous K-wire fixation shows higher overall infection rates than buried wires but does not increase deep surgical site infections requiring operative revision. Considering the avoidance of additional anesthesia, favorable cost-benefit profile, and geographic variation in infection risk, epicutaneous K-wires are recommended when no patient-specific contraindications exist, particularly in temperate climates. Level III (Systematic Review of Level II-III Studies).

## Introduction

Fractures of the upper extremity are among the most common injuries in pediatric patients, accounting for 70–80% of all fractures in children [1, 2]. Although many pediatric fractures can be managed non-operatively, surgical intervention is necessary in cases of significant fracture displacement or vascular compromise [3, 4]. Depending on the type and location of the fracture, various osteosynthesis methods exist. However, Kirschner wire (K-wire) fixation is considered as one of the standard techniques, particularly for displaced upper extremity fractures [5, 6]. Following K-wire fixation, surgeons face a clinical decision: whether to bury the wire ends subcutaneously or leave them exposed epicutaneously. To date, no definitive consensus exists on this matter. Leaving K-wire ends epicutaneous offers several advantages, including cost-effectiveness and elimination of the need for a second anesthesia for implant removal. Conversely, concerns about higher infection risk and potential psychological trauma to children from visible wires are compelling arguments against this approach.

The question of optimal K-wire management has been addressed by several systematic reviews in recent years. Wormald et al. (2017) examined lateral condyle elbow fractures specifically, finding higher infection rates with exposed wires [7]. Chen et al. (2020) reported a pooled relative risk of 0.49 favoring buried wires for infection prevention in hand and forearm fractures [8]. More recently, Pullan et al. (2025) published a comprehensive systematic review in *Injury*, demonstrating significant differences in overall infection rates (9.4% exposed vs. 5.4% buried) [9]. However, these reviews have not systematically examined geographic or climatic factors that may influence infection rates, nor have they consistently differentiated between superficial and deep infections requiring operative intervention.

The purpose of this systematic review and meta-analysis is to provide an updated synthesis of the evidence comparing buried versus exposed K-wire fixation in pediatric upper extremity fractures, with particular attention to: (1) differentiation between superficial and deep infections, (2) geographic variation in infection rates, and (3) comprehensive assessment of all relevant complications including refracture, skin erosion, and secondary implant displacement.

## Materials and methods

This systematic review was conducted according to the guidelines of the Preferred Reporting Items for Systematic Reviews and Meta-Analyses checklist (PRISMA) [10]. The study protocol was registered in PROSPERO (ID: CRD42025599933), a database listing current meta-analyses, at the beginning of our literature search. From February 2025 to August 2025, a database search was carried out independently by the authors (A.F. and M.B.). Relevant studies reporting clinical outcome after Kirschner wire (K-wire) fixation in children upper extremity fractures were serached for in the databases of PubMed, MEDLINE, EmBase and the Cochrane Library.

### Search strategy

To identify relevant manuscripts, the following search algotrithm was used:

(((“Kirschner wire*” OR “K-wire*” OR “Bone wire*” OR “Kirschner wires” OR “percutaneous pin*” OR “wire fixation” OR “pin fixation“) AND (infection* OR “surgical site infection*” OR “wound infection*” OR “pin tract infection*” OR “postoperative infection*” OR “postoperative complication*“) AND (child OR children OR pediatric OR paediatric OR adolescent OR “pediatric fracture*” OR “paediatric fracture*”) AND (fracture* OR osteosynthesis OR “fracture fixation” OR “orthopedic fixation” OR “skeletal fixation”)) OR ((“Bone Wires”[Mesh] OR “Fracture Fixation”[Mesh] OR “Orthopedic Fixation Devices”[Mesh]) AND (“Fractures, Bone”[Mesh]) AND (“Child”[Mesh] OR “Adolescent”[Mesh]) AND (buried OR subcutaneous OR exposed OR percutaneous OR external OR “skin erosion“) AND (“Surgical Wound Infection”[Mesh] OR infection)))

### Eligibility

The following inclusion criteria were applied:

**Inclusion criteria:** - Children with upper extremity fractures undergoing surgical treatment with K-wire osteosynthesis - Age under 18 years - Modified Coleman Methodology Score (mCMS) ≥60 - Studies comparing buried versus exposed K-wire techniques - Reporting of at least one outcome of interest (infection, wire migration, refracture, secondary dislocation, skin erosion)

**Exclusion criteria:** - Pathologic fractures - Biomechanical studies - Studies including adult patients - Modified Coleman score (mCMS) below 60 - Case reports or case series without comparison groups

#### Study selection

Titles and abstracts were independently screened by two reviewers (A.F. and M.B.) for relevance according to the predetermined inclusion and exclusion criteria. In cases where no abstract was available, the full text was obtained to assess the manuscript’s relevance. Reference lists of included articles were cross-referenced to ensure no suitable study was overlooked by the search algorithm. All appropriate publications were then independently analyzed for mCMS, risk of bias, and level of evidence according to the Oxford Centre for Evidence-Based Medicine [11].

#### Data extraction

The following data were systematically extracted by two independent reviewers: patient demographics, number of patients, fracture type and location, intervention details (buried vs. exposed), infection rates (overall, superficial, and deep), secondary wire migration, secondary fracture dislocation, refracture rates, and skin erosion rates. Disagreements were resolved through discussion and consensus.

#### Quality assessment and risk of bias

Methodological quality was assessed using the modified Coleman Methodology Score (mCMS), with a threshold of > 60 required for inclusion. Risk of bias was evaluated using the ROBINS-I tool for non-randomized studies and the ROB-II tool for randomized controlled trials [12, 13]. Assessment was performed independently by two reviewers, with disagreements resolved through consensus.

#### Outcome measures

**Primary outcome:** - Overall infection rate (superficial and deep combined)

**Secondary outcomes:** - Deep surgical site infection rate (requiring operative intervention) - Superficial infection rate (managed with local care or oral antibiotics) - Secondary wire migration - Refracture rate - Secondary fracture dislocation - Skin erosion rate

#### Statistical analysis

Statistical analysis was performed using Review Manager 5.4 (RevMan 5.4, The Nordic Cochrane Centre, The Cochrane Collaboration, Copenhagen, 2014). Dichotomous outcomes were analyzed using odds ratios (OR) with 95% confidence intervals (CI). Statistical heterogeneity was assessed using the I^2^ statistic, with I^2^ > 50% indicating substantial heterogeneity. Random-effects models were applied when heterogeneity was high (I^2^ > 50%), and fixed-effects models were used when heterogeneity was low (I^2^ ≤ 50%). Results were visualized using forest plots. Subgroup analyses were performed to explore geographic variation in infection rates. Statistical significance was set at *p* < 0.05.

## Results

### Study selection

The search algorithm identified 1,900 potentially suitable manuscripts. No additional studies were identified through cross-referencing. After removing duplicates and off-topic manuscripts, studies were analyzed by abstract and full text according to eligibility criteria. Twelve manuscripts met the inclusion criteria and were included in the qualitative and quantitative analysis [5, 6, 14–22].

**Fig. 1:**
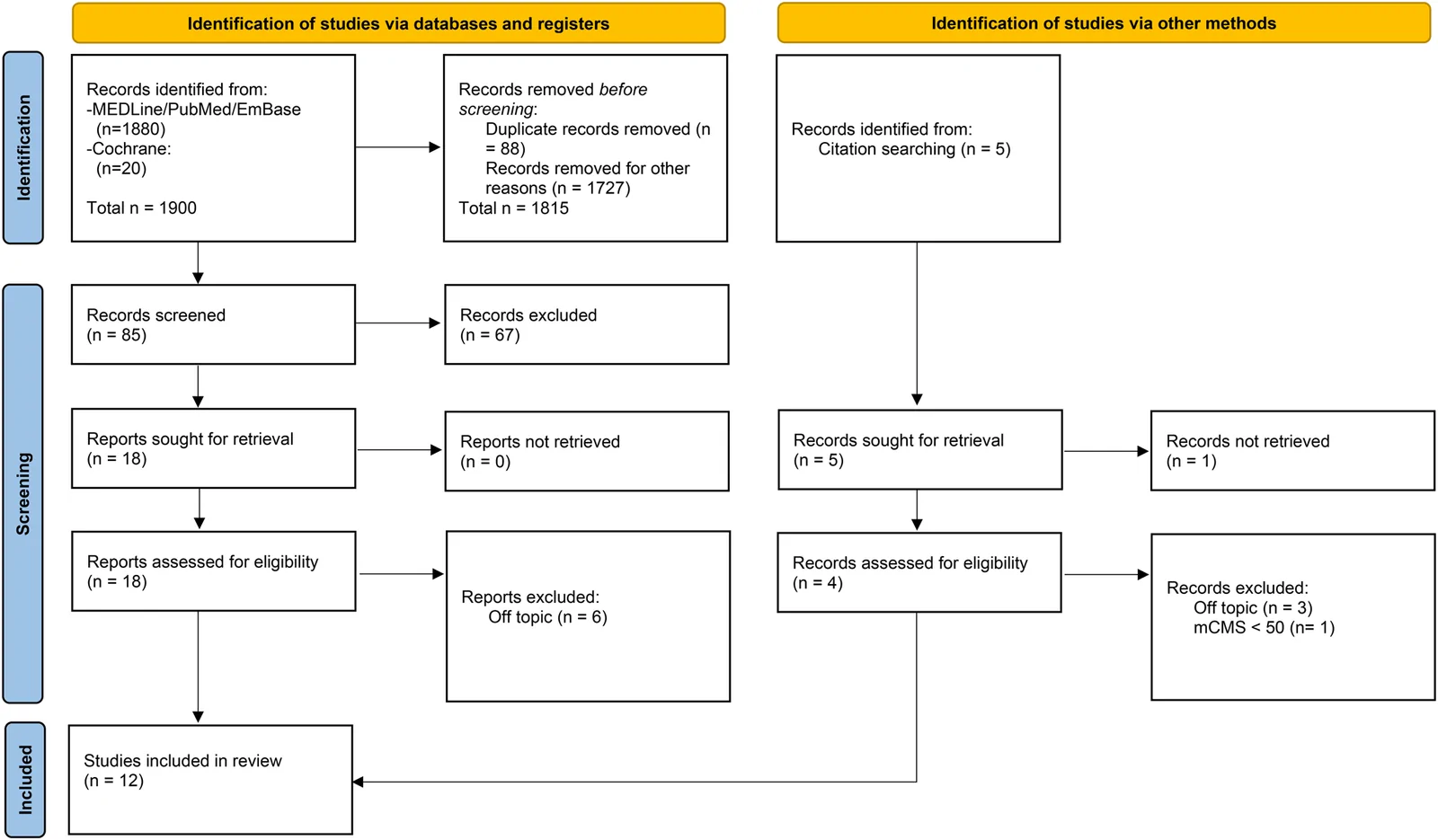
Study selection process according to PRISMA

The study selection process ist depicted in Fig. 1.

#### Study characteristics

Table 1 presents the demographic overview and characteristics of the 12 included studies, encompassing 1,977 patients. The studies were published between 2012 and 2024, with sample sizes ranging from 48 to 349 patients. Eight studies were retrospective cohort designs, three were prospective cohort studies, and one was a randomized controlled trial. Fracture types included supracondylar humerus fractures, lateral condyle fractures, and forearm fractures. The mean age of patients ranged from 5.2 to 8.7 years across studies.

**Table 1:** Overview of study demographics

Study	Studytype	Level of Evidence	Number of Patients	Gender ratio m:f	Age (Years)	Type of Surgery	Infektions	Follow Up (month)	Fracture Type
Khaled et.al. 2023	RCT	II	60	44:16	5–10	S: 30 E: 30	S: 2 E: 2	12	UA
McGonagle et.al. 2012	R	III	67	48:19	1–17	S: 55 E: 12	S: 6 E: 2	4,3	SKH
Ormsby et.al. 2016	R	III	124	80:44	5–10	S: 60 E: 64	S: 9 E: 8	5	SKH
Das De et.al. 2012	R	III	235	N.I.	Ø 6,1	S: 194 E: 42	S: 6 E: 4	4–5	SKH
Schneidemüller et.al. 2022	PnrK	III	291	N.I.	Ø 8,5	S: 167 E: 124	S: 1 E: 1	N.I.	DR, SKH
Suganuma et.al. 2022	R	III	149	86:63	1–13	S: 104 E: 45	S: 5 E: 8	N.I.	SKH
Sahoo et.al. 2022	PnrK	II	80	47:33	<12	S:40 E: 40	S: 7 E: 15	3	SKH
Prasad et.al. 2018	PnrK	II	50	37:13	1,5–11	S: 25 E: 25	S: 3 E: 7	3	SKH
Oshima et.al. 2023	R	III	143	86:63	1–13	S:79 E: 64	S: 3 E: 7	6	SKH
Kelly et.al. 2024	R	III	339	203:136	1–18	S: 208 E: 128	S: 7 E: 3	5,2	UA
Fadgyas et.al. 2024	R	III	427	273:154	1–17	S: 138 E: 289	S: 5 E: 16	N.I.	OE
Chan et.al. 2011	R	III	75	47:28	<16	S: 42 E: 33	S: 0 E: 1	N.I.	SKH

PnrK: prospektive non randomized Cohort study, RCT: randomized controlled study, R: retrospective study, S: subcutaneous K-Wire, E: epicutaneous K-Wire, DR: distael radius fracture, SKH: supracondylar humeral fracture, UA: forearm, OE: upper arm, N.I.: no information

#### Assessment of methodology and risk of bias

**Fig. 2:**
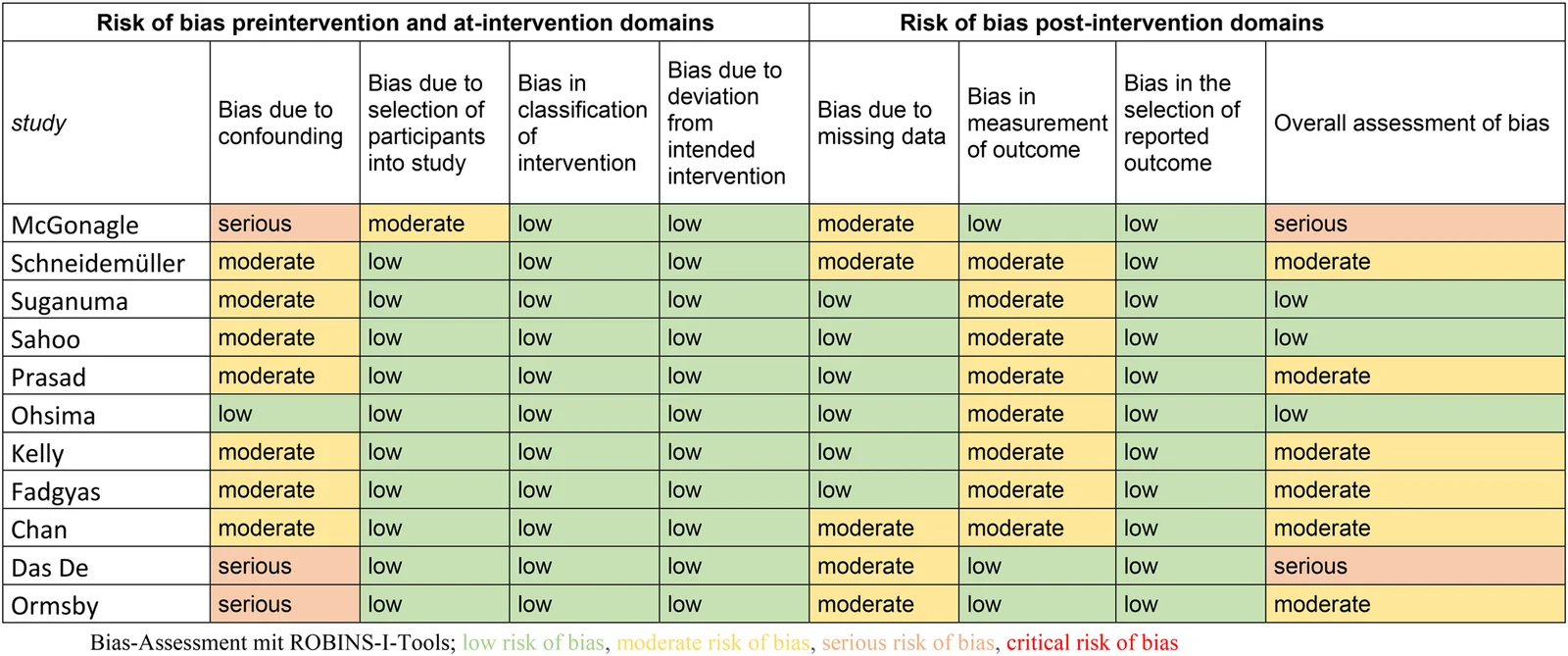
Risk of bias assessment [*Note: summary of risk of bias across all included studies using ROBINS-I and ROB-II tools]*

Most included studies possessed an evidence level of III, reflecting their retrospective design. There is a high risk of selection bias inherent in these retrospective studies. Reporting and detection biases are considerable due to the lack of randomization and blinding in most studies. However, four papers demonstrated evidence level II or higher, comprising three prospective non-controlled investigations and one randomized controlled trial (RCT). Surgical techniques were consistently reported across all studies, minimizing the risk of performance bias even when multiple surgeons were involved. Risk of bias assessment using the ROBINS-I tool for non-randomized studies and the ROB-II tool for RCTs is presented in Fig. 2.

### Wiremigration

**Fig. 3:**
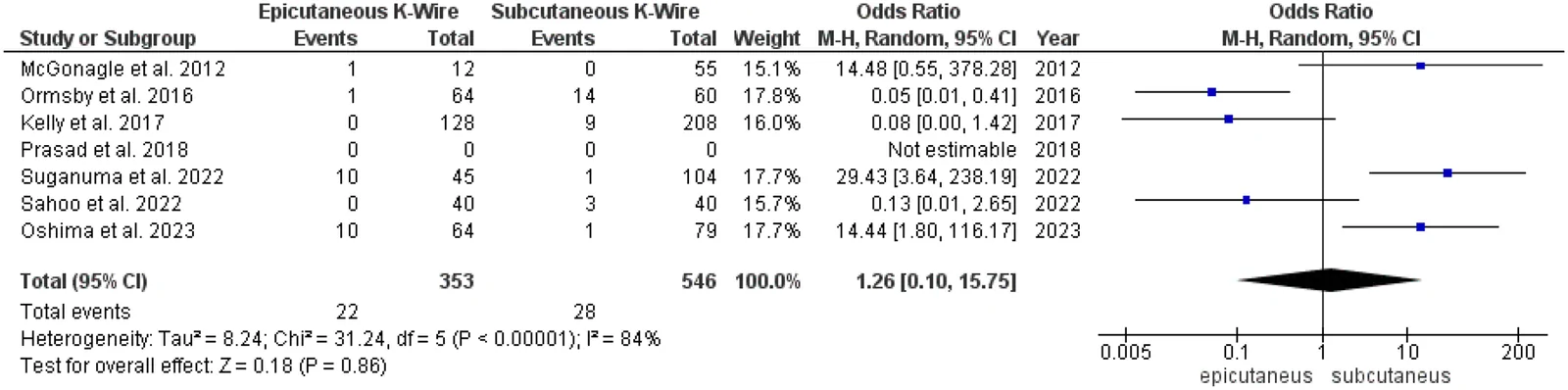
Forest plot: wire migration [*Forest plot showing odds ratios for wire migration comparing exposed versus buried K-wires across 7 studies. Overall effect: OR = 1.26 (95% CI: 0.10–15.75), p = 0.86, I*^*2*^* = 84%*]

Secondary migration of K-wires was assessed in 7 studies with a total of 899 patients. There was no significant difference between the epicutaneous and subcutaneous groups (OR = 1.26; 95% CI: 0.10–15.75; *p* = 0.86). Due to high heterogeneity of data, a random-effects model was applied (I^2^ = 84%) (Fig. 3).

#### Secondary fracture dislocation and refacture

Data on 628 patients with 26 cases of secondary fracture dislocation were analyzed. Secondary dislocation occurred in 11 patients in the epicutaneous group (*n* = 237) and 15 patients in the subcutaneous group (*n* = 391). The calculated OR was 1.27 (95% CI: 0.56–2.86; *p* = 0.56), which was not statistically significant.

**Fig. 4:**
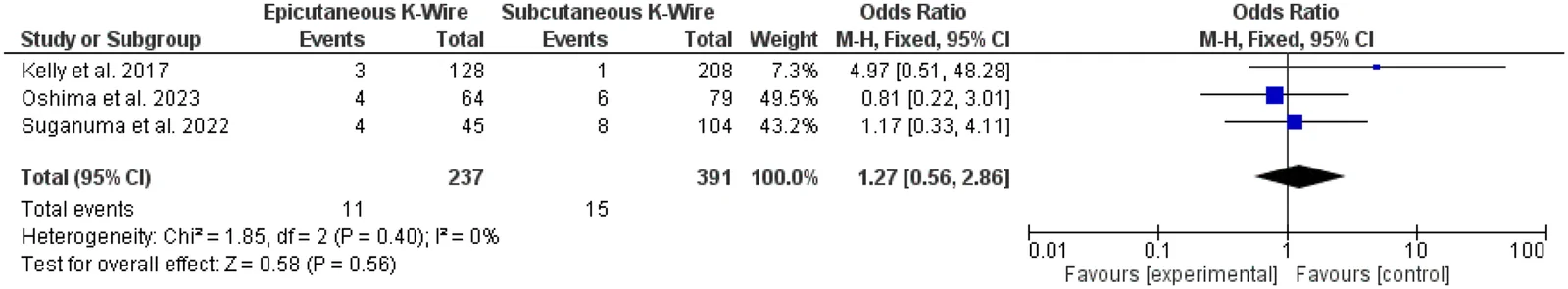
Forest plot: secondary fracture dislocation and refracture [*Forest plot showing odds ratios for secondary dislocation and refracture comparing exposed versus buried K-wires. Secondary dislocation: OR = 1.27 (95% CI: 0.56–2.86), p = 0.56. Refracture: OR = 1.90 (95% CI: 0.91–3.96), p = 0.09*]

Refractures occurred in 30 patients across 539 patients. Of these, 17 were documented in the exposed K-wire group (*n* = 222) and 13 in the buried K-wire group (*n* = 317). Due to data homogeneity, a fixed-effects model was applied to calculate the odds ratio. Although a trend was observed suggesting higher likelihood of refracture in the epicutaneous group (OR = 1.90; 95% CI: 0.91–3.96; *p* = 0.09), results did not reach statistical significance (Fig. 4).

### Overall infection rate

**Fig. 5:**
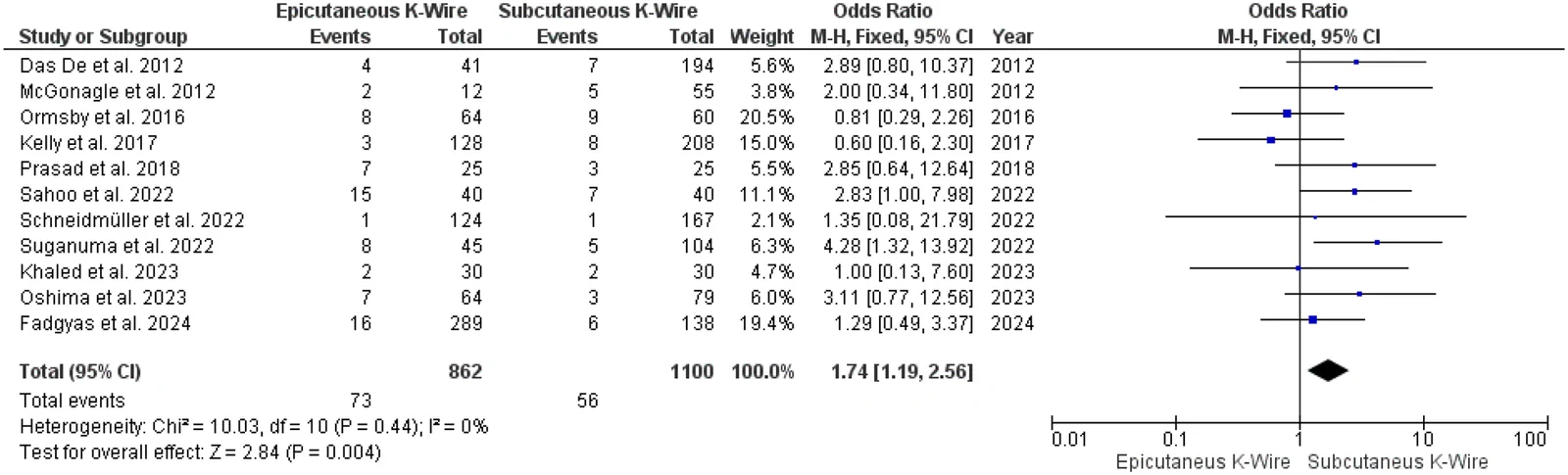
Forest plot: overall infection rate [*Forest plot showing odds ratios for overall infection rate comparing exposed versus buried K-wires across 11 studies. Overall effect: OR = 1.74 (95% CI: 1.19–2.56), p = 0.004, I*^*2*^* = 0%*]

Postoperative infections were assessed in 11 manuscripts encompassing 1,962 patients, with a total of 129 cases of superficial and deep wound infections. Of these, 73 infections (8.4%) occurred in the epicutaneous group and 56 infections (5.1%) in the subcutaneous group. Heterogeneity of data was low (I^2^ = 0%). There was a significantly lower infection rate when K-wires were buried compared to leaving ends epicutaneous (OR = 1.74; 95% CI: 1.19–2.56; *p* = 0.004) (Fig. 5).

#### Subgroup analysis of deep and superficial infections

**Fig. 6:**
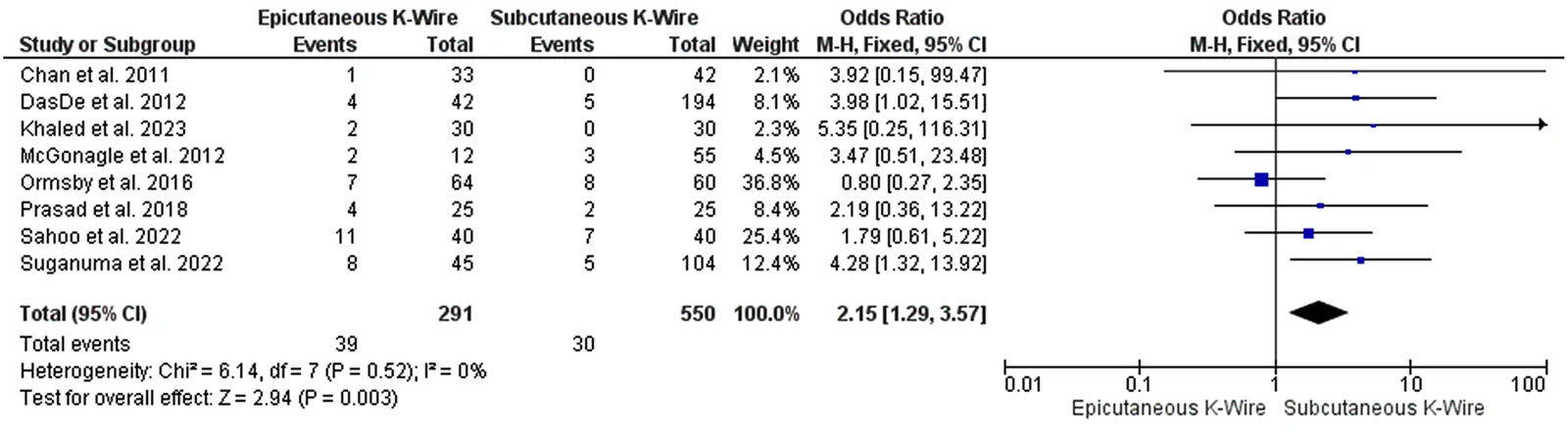
Forest plot. Superficial infection rate. [*Forest plot showing odds ratios for superficial infection rate comparing exposed versus buried K-wires across 8 studies. Overall effect: OR = 2.15 (95% CI: 1.29–3.57), p = 0.003, I*^*2*^* = 0%*]

**Fig. 7:**
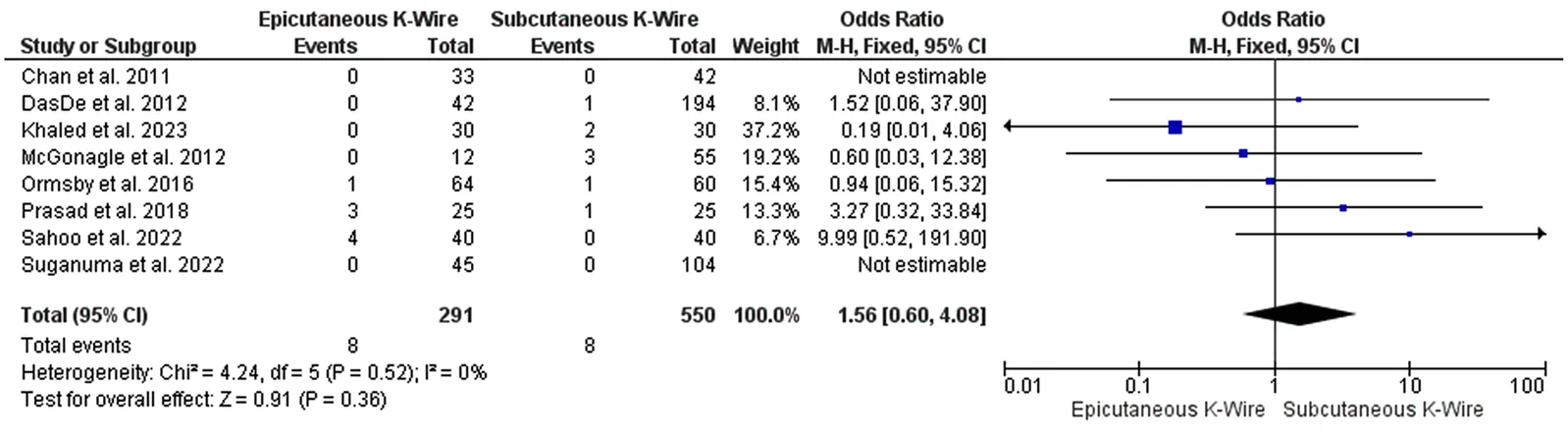
Forest plot. Deep infection rate. [*Forest plot showing odds ratios for deep wound infection rate comparing exposed versus buried K-wires across 8 studies. Overall effect: OR = 1.56 (95% CI: 0.60–4.08), p = 0.36, I*^*2*^* = 0%*]

To provide a more detailed assessment of postoperative infection rates, a subgroup analysis was performed according to the depth of infection, distinguishing between deep and superficial postoperative infections. In eight of the included manuscripts, postoperative infections were further classified as either deep or superficial. The results of these subgroup analyses are presented in Figs. 6 and 7.

Across all studies reporting superficial infections, these were managed conservatively with antibiotic therapy. In contrast, deep infections were managed surgically, with debridement followed by antibiotic therapy.

Across the eight studies, a total of 69 superficial pin-tract infections were reported. Of these, 39 (13.4%) occurred in patients with epicutaneous K-wires, compared with 30 (5.5%) in patients with subcutaneous K-wires. Heterogeneity among the included studies was low, with an I^2^ value of 0%. There was a significantly higher rate of superficial wound infections when k-wires were left unburied compared to the group where the ends have been buried (OR = 2.15; 95% CI: 1.29–3.57; *p* = 0.003). (Fig. 6)

Regarding deep wound infections, eight cases were reported in each group, corresponding to an infection rate of 2.7% in the epicutaneous K-wire group and 1.4% in the subcutaneous K-wire group. Neither group demonstrated a statistically significant increased risk of deep wound infection (Fig. 7).

#### Geographic subgroup analysis

To investigate potential confounding by regional differences, a subgroup analysis was performed dividing study data by geographic region. Significant differences between epicutaneous and subcutaneous infection rates were observed in studies performed in Asia and Africa, while no difference was detected in European countries and the USA. These regions were therefore grouped and analyzed separately.

**Fig. 8:**
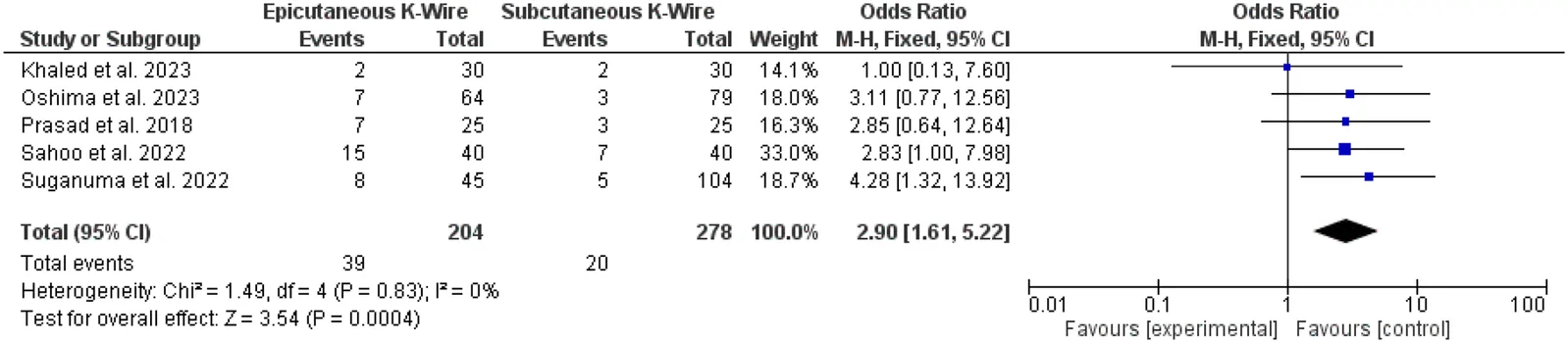
Forest plot: infection rate in Asia/Africa [*Forest plot showing odds ratios for infection rate in Asia/Africa subgroup. Overall effect: OR = 2.90 (95% CI: 1.61–5.22), p = 0.0004*]

**Asia/Africa Subgroup** (Fig. 8): Five studies with a total of 482 patients were conducted in Asia or Africa. Infections occurred in 39 patients (19.1%) with epicutaneous K-wires and 20 patients (7.2%) with subcutaneous K-wires. The OR was significant at 2.90 (95% CI: 1.61–5.22; *p* = 0.0004).

**Fig. 9:**
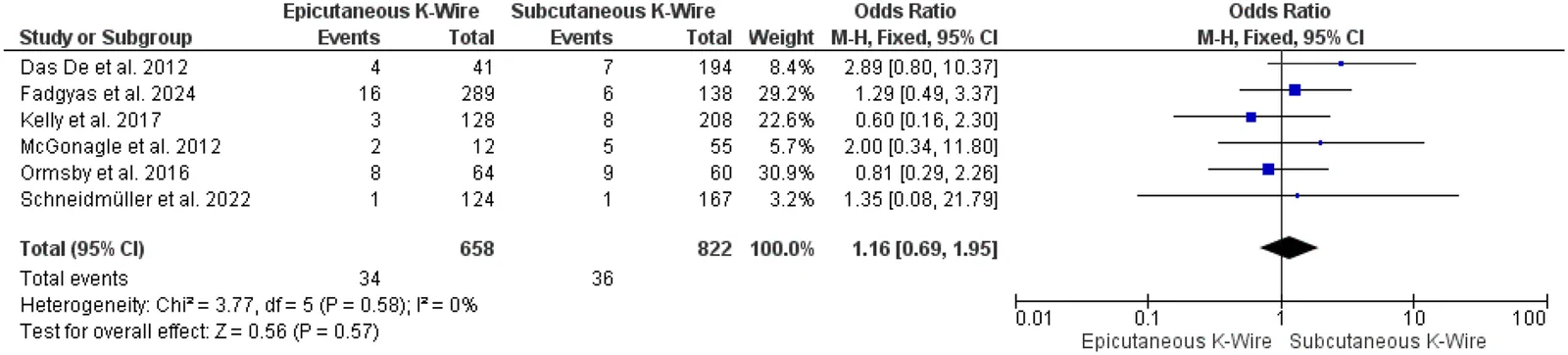
Forest plot: infection rate in Europe/USA [*Forest plot showing odds ratios for infection rate in Europe/USA subgroup. Overall effect: OR = 1.16 (95% CI: 0.69–1.95), p = 0.57*]

**Europe/USA Subgroup** (Fig. 9): Six studies encompassing 1,480 patients were conducted in the USA or Europe, with 36 postoperative infections (4.4%) documented when wires were buried (*n* = 822) and 34 infections (5.2%) with epicutaneous wire ends (*n* = 658). The OR was calculated as 1.16 (95% CI: 0.69–1.95; *p* = 0.57), showing no significant difference between intervention groups.

### Skinerosion

**Fig. 10:**
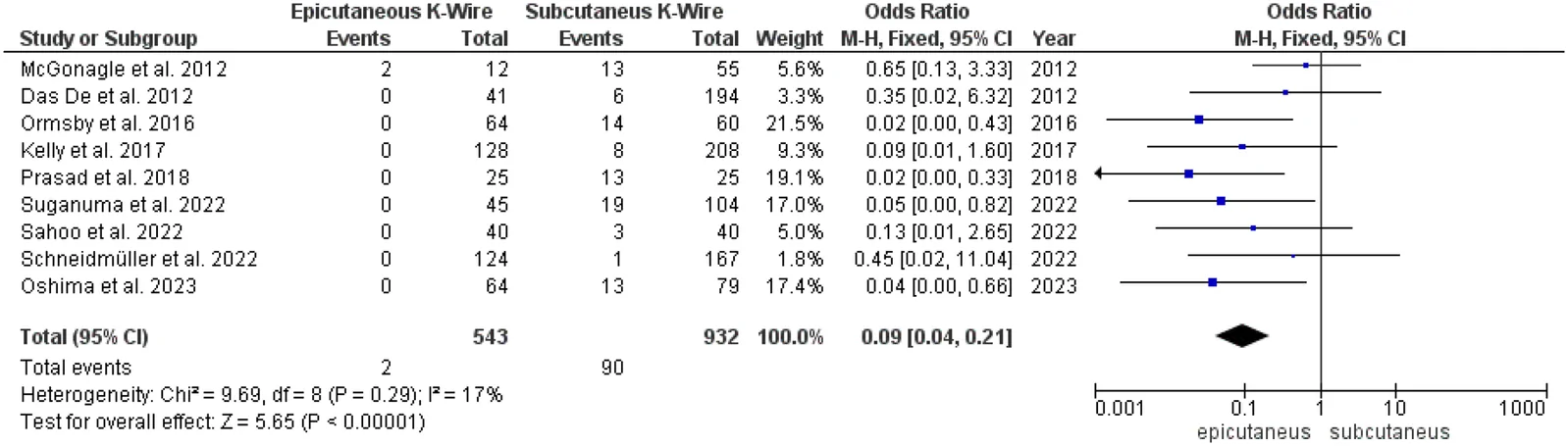
Forest plot: skin erosion [*Forest plot showing odds ratios for skin erosion comparing exposed versus buried K-wires. Overall effect: OR = 0.09 (95% CI: 0.04–0.21), p < 0.00001*]

Skin erosion can occur when long wire ends are buried too superficially beneath the skin or when wires are placed under tension. We analyzed the rate of skin erosion for both epicutaneous and subcutaneous K-wires. Only two cases were observed in the exposed K-wire group, whereas 90 patients experienced skin erosion in the subcutaneous group. These findings were statistically significant (OR = 0.09; 95% CI: 0.04–0.21; *p* < 0.00001) (Fig. 10).

## Discussion

The principal findings of this systematic review and meta-analysis demonstrate that burying K-wires subcutaneously leads to a significant reduction in overall postoperative infections compared to leaving wires exposed epicutaneously. However, when infections are stratified by severity, no significant difference exists in deep surgical site infections requiring operative intervention. Additionally, no significant differences were observed in rates of refracture or secondary wire migration. Notably, our geographic subgroup analysis revealed substantial regional variation, with infection rate differences being significant in Asia and Africa but not in Europe and the USA.

### Infection rates: overall versus deep infections

The question of whether K-wire ends should be buried subcutaneously or left exposed epicutaneously remains a subject of ongoing debate in pediatric orthopedic surgery. A primary concern with epicutaneously exposed K-wires is that they may serve as a portal of entry for bacteria and facilitate biofilm formation. However, reported rates of postoperative infection vary considerably across the literature [23]. Our meta-analysis confirms findings from recent systematic reviews, including Pullan et al. (2025), who reported infection rates of 9.4% for exposed wires versus 5.4% for buried wires [9]. Similarly, Chen et al. (2020) demonstrated a pooled relative risk of 0.49 favoring buried wires [8]. Our analysis extends these findings by demonstrating an overall infection OR of 1.74 (95% CI: 1.19–2.56; *p* = 0.004) favoring buried wires.

Critically, however, a major limitation in the available literature is the frequent lack of differentiation between deep surgical site infections requiring operative intervention and superficial infections not necessitating surgical treatment. In the eight studies included in our meta-analysis that applied a more detailed classification of infections, no significant differences were observed in deep surgical site infections between exposed and buried K-wire fixation (*OR = 1.56 (95% CI: 0.60–4.08), p = 0.36*). This finding suggests that skin integrity or the presence of osteosynthesis material traversing the skin does not appear to exert a decisive influence on the incidence of deep infections requiring operative revision. Superficial infections associated with exposed wires can generally be managed with local wound care or oral antibiotic therapy, avoiding the need for operative intervention. Among the included studies deep wound infections were treated consistently across all studies included in this meta-analysis. Treatment consisted of surgical debridement followed by intravenous antibiotic therapy. Superficial wound infections were consistently managed with oral antibiotic therapy.

### Geographic and climatic influences

A novel finding of our analysis is the identification of significant geographic variation in infection rates. Studies conducted in Asia and Africa demonstrated a substantial difference in infection rates between exposed and buried wires (OR = 2.90; 95% CI: 1.61–5.22; *p* = 0.0004), whereas studies from Europe and the USA showed no significant difference (OR = 1.16; 95% CI: 0.69–1.95; *p* = 0.57).

Possible geographic and climatic factors influencing pin-tract infections in children were investigated by Kao et al., who demonstrated that warmer and more humid climates are associated with a significant increase in infection rates [24]. Their study provides a plausible mechanistic explanation for our observed geographic differences. However, it is important to acknowledge that geographic variation may also reflect differences in healthcare resources, antibiotic availability, infection surveillance intensity, reporting practices, and pin-care protocols rather than climate alone. Regarding the pin-care protocols, the individual studies differed in terms of the timing and number of follow-up visits, particularly during the first few postoperative weeks. However, consistent features across all studies included immobilization using a backslab and scheduled follow-up visits at 2 and 6 weeks postoperatively. None of the included studies provided a detailled pin-care protocol or recommendations given to patients how often wound dressings have to be changes in between the follow-up visits.

The ecological nature of this subgroup analysis, based on continent-level grouping, limits our ability to definitively attribute observed differences to climate. Furthermore, the Asia/Africa subgroup comprised only 5 studies with 482 patients, limiting generalizability.

Despite these limitations, our findings suggest that surgeons practicing in warmer, more humid climates should more strongly consider buried K-wire fixation, whereas those in temperate climates may safely employ exposed wires in most cases. This represents hypothesis-generating evidence that warrants validation through prospective studies incorporating direct measurement of local climate conditions and standardized pin-care protocols.

### Avoidance of secondary anaesthesia and surgery

A major argument in favor of epicutaneous K-wire fixation is the avoidance of a second surgical procedure for wire removal. This eliminates an otherwise avoidable anesthetic risk and reduces additional operative risks. Although the overall incidence of anesthesia-related complications in children undergoing elective surgery is low, any avoidable exposure to general anesthesia represents a meaningful benefit, particularly when multiplied across the large number of pediatric fractures requiring K-wire fixation annually [25].

### Pain and psychological considerations

Pain or psychological distress associated with cleaning of exposed K-wires, as well as emotional stress during wire removal, are frequently cited as reasons for burying K-wires subcutaneously [26]. Pain perception during wire removal was evaluated by Sorenson et al. using the Children’s Hospital of Eastern Ontario Pain Scale (CHEOPS) in children with supracondylar humerus fractures [27]. The CHEOPS score ranges from a minimum of 4 to a maximum of 13 points. In their analysis of 98 patients, the mean score was 6 points, corresponding to no or only mild pain during K-wire removal. Thus, pain itself does not appear to be a major limiting factor for outpatient wire removal; rather, individual anxiety of the patient and their caregivers plays a more substantial role.

When comparing both techniques, the anxiety and discomfort associated with outpatient removal of epicutaneously exposed K-wires must be weighed against the anxiety and logistical burden of the subcutaneous technique. The prospect of general anesthesia and hospital admission may likewise be distressing for a child. Consequently, consistent and empathetic counseling of both patients and their caregivers plays a crucial role in reducing anxiety and facilitating shared decision-making.

### Cost-effectiveness considerations

Cost-effectiveness represents an important consideration in healthcare resource allocation. Outpatient removal of epicutaneous K-wires requires only basic instruments (e.g., pliers), dressing materials, a brief physician visit, and, if necessary, simple analgesics. In contrast, removal of subcutaneously buried wires necessitates comprehensive operative preparation, operating theater availability, surgical and anesthesia personnel, and at least a short inpatient stay. Several studies have highlighted the cost-effectiveness of epicutaneous K-wire fixation, with estimated cost savings ranging from USD 800 to 3,500 per patient [14, 26, 28]. When multiplied across the thousands of pediatric upper extremity fractures treated annually, these savings represent substantial healthcare resource conservation.

However, a complete cost-effectiveness analysis must also consider the costs associated with treating the higher rate of superficial infections observed with exposed wires. While most superficial infections can be managed with oral antibiotics and local wound care, some may require additional clinic visits, cultures, and occasionally intravenous antibiotics. Future research incorporating formal cost-effectiveness modeling with calculation of incremental cost-effectiveness ratios would provide more definitive guidance.

### Comparison to existing literature

Our findings are consistent with and extend the existing body of systematic reviews on this topic. Wormald et al. (2017) examined lateral condyle fractures specifically and found higher infection rates with exposed wires [7]. Raghavan et al. (2019) concluded that exposed wires are an acceptable, economical option despite higher superficial infections [29]. Hidajat et al. (2024) conducted a comprehensive meta-regression on hand and forearm fractures, demonstrating significantly lower infection rates with buried wires [30]. Most recently, Pullan et al. (2025) published a systematic review in *Injury* showing infection rates of 9.4% for exposed versus 5.4% for buried wires [9].

Our analysis contributes to this literature by: (1) providing updated pooled estimates incorporating recent studies through 2024–2025, (2) systematically examining geographic variation in infection rates, (3) emphasizing the distinction between superficial and deep infections, and (4) comprehensively assessing multiple outcomes including wire migration, refracture, and skin erosion in a single analysis. The geographic subgroup analysis represents the most novel contribution, providing hypothesis-generating evidence that climate and regional factors may influence the relative safety of exposed versus buried K-wire fixation.

### Limitations

This study has several limitations that warrant acknowledgment. First, the literature search was restricted to English- and German-language publications, potentially excluding relevant studies in other languages. Second, the inclusion of only published studies introduces a risk of publication bias, although our assessment of heterogeneity and funnel plot analysis (not shown) did not suggest substantial publication bias.

Third, most included studies were retrospective case-control or cohort designs, which are inherently prone to selection, detection, and reporting bias. Only one randomized controlled trial was included. To address this limitation, we assessed methodological quality using the modified Coleman Methodology Score (mCMS > 60 threshold) and risk of bias using ROBINS-I and ROB-II tools. No study demonstrated a critical risk of bias.

Fourth, infection definitions varied across studies, with only 8 of 11 studies differentiating between superficial and deep infections. This heterogeneity in outcome definition limits the precision of our estimates. Future studies should employ standardized infection definitions, ideally based on validated criteria such as those proposed by the Centers for Disease Control and Prevention.

Fifth, the geographic subgroup analysis, while novel, is based on a relatively small number of studies (5 studies, 482 patients in the Asia/Africa subgroup) and employs continent-level grouping that may mask within-region variation or differences in post surgery wound care. The observed differences may reflect confounding by healthcare resources, antibiotic availability, infection surveillance practices, and reporting standards rather than climate alone. This analysis should be considered hypothesis-generating rather than definitive.

Sixth, we were unable to conduct subgroup analyses by fixation duration, fracture type, age groups, or pin-care protocols due to insufficient reporting of these variables across studies. Future systematic reviews would benefit from individual patient data meta-analysis to enable more granular subgroup analyses.

Finally, the refracture analysis showed a trend toward higher rates with exposed wires (OR = 1.90; *p* = 0.09) that approached but did not reach statistical significance. With only 30 events across 539 patients, this analysis may have been underpowered to detect a clinically meaningful difference, representing a potential Type II error.

### Clinicaal implications and recommendations

Based on our findings, we propose the following clinical recommendations:

1. **General recommendation:** Epicutaneous K-wire fixation is a safe and cost-effective option for most pediatric upper extremity fractures, particularly in temperate climates.
2. **Geographic considerations:** Surgeons practicing in warm, humid climates (tropical and subtropical regions) could consider buried K-wire fixation instead of unburied K-wire ends as warm climates seem to affect infection rates with exposed wires in these settings.
3. **Infection risk stratification:** The higher overall infection rate with exposed wires is driven primarily by superficial infections that can be managed non-operatively. Deep infections requiring surgical intervention occur at similar rates regardless of wire burial status.
4. **Patient-specific factors:** Buried wires should be considered in patients with:
  Compromised immune function
  Poor hygiene or unreliable follow-up
  High anxiety about visible wires
  Residence in warm, humid climates
  Complex fractures requiring prolonged fixation
5. **Shared decision-making:** Surgeons should engage in shared decision-making with families, discussing the trade-offs between slightly higher superficial infection risk with exposed wires versus the need for second anesthesia with buried wires.
6. **Pin-care protocols:** Regardless of burial status, standardized pin-care protocols should be employed, with consideration of climate-adapted regimens in warm, humid environments.

### Future research directions

Several important research questions remain unanswered and warrant future investigation:

1. **Prospective multicenter RCTs:** Large, well-designed randomized controlled trials comparing buried versus exposed K-wires with standardized infection definitions and adequate power for subgroup analyses.
2. **Climate-adapted protocols:** Prospective studies examining the interaction between climate conditions and pin-care protocols, building on the work of Kao et al. [24].
3. **Patient-reported outcomes:** Systematic assessment of pain, anxiety, satisfaction, and quality of life using validated instruments.
4. **Cost-effectiveness modeling:** Formal decision-analytic models incorporating infection rates, treatment costs, quality-adjusted life years, and probabilistic sensitivity analyses.
5. **Predictive models:** Development and validation of clinical decision rules to identify patients at highest risk for complications with exposed wires.
6. **Long-term outcomes:** Assessment of long-term functional outcomes, including range of motion, strength, and return to activity.

## Conclusion

Epicutaneous K-wire fixation is associated with a higher overall infection rate compared with subcutaneously buried wires. However, no significant difference is observed with regard to deep surgical site infections, and consequently no increased need for operative revision is evident. Superficial infections can generally be managed with local treatment or antibiotic therapy. Geographic variation exists, with infection rate differences being significant in warm, humid climates (Asia/Africa) but not in temperate regions (Europe/USA).

When weighing the additional risks of anesthesia and surgery required for wire removal, as well as the substantial cost savings (USD 800–3,500 per patient), we recommend the use of epicutaneously exposed K-wires as the preferred approach for most pediatric upper extremity fractures, provided that no patient-specific factors contraindicate their use. In warm, humid climates, surgeons should more strongly consider buried K-wire fixation due to higher infection rates with exposed wires in these settings. Shared decision-making with families, incorporating discussion of infection risk, cost, and need for second anesthesia, should guide individualized treatment decisions.

## Data Availability

No datasets were generated or analysed during the current study.
